# Predictors of vaccination card retention in Tamale Metropolis, Ghana

**DOI:** 10.1371/journal.pone.0292765

**Published:** 2024-02-26

**Authors:** Matthew Y. Konlan, Fuseini Mahama, Braimah B. Abubakari, Paul Konka, Benedict O. Appiah, Maxwell O. Yeboah, Peter G. Kwarteng, Porbilla O. Apea, Michael R. Adjei, Martin N. Adokiya, Oheneba Boadum, Hilarius A. K. Abiwu

**Affiliations:** 1 Northern Regional Health Directorate, Ghana Health Service, Tamale, Ghana; 2 Department of Nursing, Northern Regional Hospital, Ghana Health Service, Tamale, Ghana; 3 UNICEF, Northern Zonal Office, Tamale, Ghana; 4 WHO Ghana Country Office, Accra, Ghana; 5 Department of Epidemiology, Biostatistics and Disease Control, School of Public Health, University for Development Studies, Tamale, Ghana; 6 Department of Advanced Biomedical Education, University of Mississippi Medical Center, Jackson, MS, United States of America; University for Development Studies, GHANA

## Abstract

**Background:**

The home-based vaccination card is an important health record for determining vaccination status of children during surveys, particularly in low- and middle-income countries. However, there are limited evidence on the factors that influence its retention in Ghana. We assessed the predictors of vaccination card retention in Tamale Metropolis, Ghana.

**Methods:**

We conducted a cross-sectional study from 21^st^ December 2022 to 10^th^ January 2023 among children aged 0–59 months in the Tamale Metropolis. Multi-stage sampling was used to select caregivers of children aged 0–59 months for enrolment in the study. Data were collected using validated questionnaire through face-to-face interviews of caregivers. A vaccination card was retained if it was presented for physical inspection by research assistants. The factors that influence vaccination card retention were determined in a multivariate logistic regression analysis at p<0.05.

**Results:**

A total of 1,532 eligible children were enrolled in this study. Vaccination card retention was 91.5%. Negative predictors of card retention included: being resident in the Nyohini (AOR = 0.28; 95% CI = 0.15–0.50) and Tamale Central (AOR = 0.51; 95% CI = 0.29–0.90) sub-Metro areas and being caregivers of children aged 24–59 months (AOR = 0.39; 95% CI = 0.22–0.68). On the other hand, paying for the vaccination card (AOR = 5.14; 95% CI = 2.95–8.95) was a positive predictor of vaccination card retention.

**Conclusion:**

In this study, vaccination card retention among children aged 0–59 months was higher than national estimates. Vaccination card retention was mainly influenced by sub-Metro area, age of child and mode of acquisition of the card such as out-of-pocket payment. There is need to design and deliver tailored messages including the importance of vaccination card retention to caregivers of children based on geographic context. Additionally, the policy on sale of vaccination cards should be revised to allow for cost sharing to enhance its retention.

## Introduction

Routine childhood vaccination is one of the most successful public health interventions, averting four million deaths every year [1]. Aside protection against preventable diseases, vaccination brings children and their families into contact with health systems, providing an opportunity for delivery of other health services [1]. Routine immunization reduces vaccine-preventable disease incidence [2], antimicrobial use [3], and resistance [4] and associated deaths [5]. Ghana’s childhood vaccination schedule, which targets children under five years has consistently changed since the adoption of the Expanded Program of Immunization (EPI). The most recent schedule targets different cohorts. Currently, DPT is not given to infants in Ghana as a stand-alone vaccine. Instead, it has been combined with other antigens that protect against hepatitis B and type b Haemophilus influenzae, and this vaccine (DPT-HepB-Hib) is known as the pentavalent vaccine. The Rota virus vaccine was previously administered in two doses, one month apart until in 2020 when the 3rd dose was included in the schedule. In the routine EPI schedule in Ghana, BCG and OPV0 are given at birth, OPV, Penta (DPT, HepB, Hib), PCV, and Rota are given at the 6^th^, 10^th^, and 14^th^ weeks of birth. IPV is given at the 14^th^ week of birth, the Measles-Rubella vaccine is given at the 9^th^ and 18^th^ months of birth, the yellow fever and meningitis A vaccines are given at the 9^th^ and 18^th^ months respectively [6].

Vaccination services are delivered freely to all eligible recipients including issuance of vaccination cards at first vaccination. Vaccination data are collected during service delivery through vaccination registers and individual home-based vaccination cards (HBRs). The vaccination registers are paper-based records kept in the facility and used for estimating administrative coverages while the HBRs, which records child’s age with corresponding vaccines given at that age, the date vaccines are given, the batch number of vaccines, place vaccine is given and by who, and the next due date of vaccines, are kept by caregivers of children for follow ups on the vaccination schedule and used as reference source document for vaccination coverage surveys. Often, these HBRs get lost or destroyed. Meanwhile, in low- and middle-income countries (LMICs), HBRs are the primary means for assessing vaccination status of children during surveys [7,8]. Additionally, HBRs serve as a medium for the delivery of reminder strategies on when to bring a child for future vaccinations [9], promoting adherence to routine childhood vaccination uptake [10]. However, they are less valued, retained and used by caregivers to support healthcare decisions [11].

Available data suggests that vaccination card retention varies across countries and particularly low in sub-Saharan Africa. A study conducted by Wagner reported retention rate of 23.3% in Nigeria, 49.6% in Angola, 29.5% in Ethiopia, and 20.7% in Democratic Republic of Congo [12]. Studies conducted in Yaounde-Cameroon [13] and Senegal [14] also reported retention rates of 24% and 86.6% respectively. While child age and maternal employment were found to be negatively associated with card retention, being a girl child, living in rich household and respondent being the biological parent of the child were positively associated with card retention in Yaounde-Cameroon [13]. In Senegal, child age and maternal exposure to the media were associated with card retention. Additionally, home delivery and infrequent antenatal care attendance were negatively associated with vaccination card retention [14].

In Ghana, the current national vaccination card retention rate is 78.5% [15], a decline from 2014 rate of 80% [16]. Understanding factors that influence vaccination card retention can help in the design and delivery of tailored messages to improve retention. However, studies on predictors of vaccination card retention are limited in Ghana. To fill this gap, and further understand factors for non-retention of vaccination cards among caregivers, this study assessed predictors of vaccination card retention among children aged 0–59 months in Tamale Metropolis, Ghana.

## Materials and methods

### Study design

This was a community-based analytical cross-sectional study conducted from 21^st^ December 2022 to 10^th^ January 2023 among children aged 0–59 months in the Tamale Metropolis. This study was carved out of a larger community-based cross-sectional pre- and post-COVID-19 pandemic cluster survey of children under five years in Tamale Metropolis.

### Study area

This study was conducted in Tamale, which doubles as the administrative capital and district capital of the Northern Region and Tamale Metropolis respectively. According to the 2021 Population and Housing Census, the Metropolis has a population of 374,744: 185,051 males and 189,693 females [17]. The Metropolis is divided into four (4) health sub-Metros: Vittin, Bilpeila, Nyohini and Tamale Central, with 77 health facilities including Community-based Health Planning and Services (CHPS), which render a range of services including vaccination. Administrative coverages of routine immunizations in Tamale have consistently been above 100% in the last five years except Rota 3 and Men A, with occasional outbreaks of vaccine preventable diseases. Tamale Metropolis was chosen to understand the extent of card retention and factors influencing vaccination card retention in urban settings to inform tailored decisions.

### Study population

Caregivers of children aged 0–59 months who were living in the Tamale Metropolis for at least six months before the study were eligible for enrolment in the survey.

### Sample size calculation

Based on the WHO survey sample size calculator version 2 [18], we estimated a sample of 1, 512 caregivers of children aged 0–59 months using the following assumptions: 95% coverage for all basic immunizations (Northern Regional target), a precision of ±5% (95% confidence interval), a design effect (DEFF) of 2, assumption of a minimum of 5 children per cluster, an intra-cluster correlation coefficient (ICC) of 1/3, assuming that 10% of eligible respondents will not be available or will decline the survey and that we would need to visit 4 households to identify an eligible child.

### Selection of respondents

Multi-stage sampling was used to select respondents. In stage one, all 63 clusters were purposively selected from the four (4) sub-Metros in Tamale. Clusters were made up of demarcated CHPS zones in the study area, and a list of all clusters and the communities under each cluster was obtained from the Tamale Metro Health Directorate. In stage two, a list of all communities in each cluster was generated and coded. Sixty-three (63) communities, one from each cluster were then selected using simple random sampling. In stage three, a random direction from the center of each community was first selected by spinning a pen. The houses along that direction were then counted out to the boundary of the community, and one house was selected randomly for enumeration of the first household. Subsequently, houses were selected using an interval of three and two in urban and peri-urban localities respectively. In stage four, simple random sampling was used to select eligible children. In households with more than one eligible child, the lottery method was used to select one child. Twenty-four (24) households were randomly selected in each cluster. All children aged 0–59 months living in each selected household was enrolled in the study.

#### Eligibility criteria

All caregivers of children aged 0–59 months were included in this study. Caregivers of children above 59 months, those who had never received a vaccination card, and those who declined were excluded. Caregivers who had lived in the study setting for less than six months before the survey were excluded.

### Data collection

Data were collected using validated questionnaire by the WHO for assessing routine immunization coverage [19]. The questionnaire was categorized into; 1) sub-Metro variables, 2) socio-demographic variables of eligible caregiver-child pairs, and 3) vaccination coverage using card and caregiver recall of vaccination questions. Questionnaire was deployed using the latest android version (v2021.2.4) of KoboCollect App [20] in Samsung mobile tablets. Trained research assistants collected data through face-to-face interviews with respondents. During the survey, caregivers were requested to present vaccination cards to confirm retention.

### Quality assurance

Research assistants were given a day’s training on survey objectives, methods and how to ask questions to elicit appropriate responses. Tools were pretested in Savelugu, a district with similar characteristics as Tamale Metropolis and modified by investigators to enhance clarity. Pictures of the biodata and vaccination record pages of eligible children were taken to confirm card retention. Submitted questionnaires were checked for correctness and completeness.

### Operational definitions

A vaccination card was defined as any document given by a healthcare provider stating the vaccination status of an eligible child [21].

A caregiver is defined as an individual who provides physical and psychological care for the child.

Card was retained if a document was seen and verified by research assistants as proof of vaccination status of eligible child.

### Data analysis

Data were exported from KoboCollect into excel for cleaning before being imported in Stata Version 15.1 (Stata Corp (2017) Stata Statistical Software: Release 15. College Station, TX.) for analysis. Card retention was the outcome variable, which was determined through correct “Yes”/ “No” responses to the physical inspection of a vaccination card presented for review of research assistants by caregivers as proof of vaccination status. The exposure variables were sub-Metro, sex of child, age of child, place of delivery, sex of caregiver, age of caregiver, employment status of caregiver, and educational status of respondent, relationship of child to respondent and whether card was paid for (out-of-pocket). Potential exposure variables were first assessed in a univariate regression analysis to determine associations and only factors that showed statical significance at P<0.35 were included in the multivariate regression model without any forward or backward selection. This allowed for more interaction among variables of interest. The predictors of vaccination card retention were assessed through a multivariate logistic regression model and the level of significance was determined at p<0.05.

### Ethical approval

This study received approval from the Navrongo Health Research Center Institutional Review Board (Approval ID: NHRCIRB495). Verbal consent was obtained from caregivers of eligible children before enrolment in the study. Study objectives, approach and rights of respondents were explained in detail and clarifying questions addressed by the research assistants.

## Results

### Sample description

Though our estimated sample size was 1512, we enrolled 1577 eligible children. However, only 1,532 were included in the data analysis after cleaning, comprising 723 (47.2%) girls and 809 (52.8%) boys, and 1452 (94.8%) female and 80 (5.2%) male caregivers. Most respondents were enrolled from the Tamale Central (27.4%) and Bilpeila sub-Metros (26.6%). The mean (Standard Deviation) age of the children was 34 (14) months, with majority (47.4%) belonging to the age group 24–59 months, followed by 0–9 months (22.3%) and 10–18 months (20.4%). Majority (95.1%) of the caregivers who were enrolled in this study were the mothers of the children, majority (90.3%) of whom were born in a publicly owned health facility. The mean (standard deviation) age of caregivers was 46 (23) years, with the majority being less than 30 years (67.6%) and unemployed (69.6%). Most of the caregivers had attained primary education (49.2%) and reportedly didn’t pay for the vaccination card (62.7%) (see Table 1).

**Table 1:** Sub-Metro type and socio-demographic characteristics of respondents and children in Tamale Metropolis, Ghana.

Variable	Frequency (%)
**Sub-Metro**	
Bilpeila	407 (26.5)
Nyohini	351 (22.9)
Tamale Central	419 (27.4)
Vittin	355 (23.2)
**Child-related characteristics**	
**Sex of child**	
Female	723 (47.2)
Male	809 (52.8)
**Age of child (months)**	
Mean (Standard Deviation)	32 (14)
0–9	341 (22.3)
10–18	313 (20.4)
19–23	152 (9.9)
24–59	726 (47.4)
**Relationship of child to respondent**	
Mother	1,457 (95.1)
Father	60 (3.9)
Other^b^	15 (1.0)
**Place of delivery of child**	
Home/TBA	102 (6.7)
Privately owned facility	47 (3.0)
Public owned facility	1,383 (90.3)
**Respondent-related characteristics**	
**Sex of respondent**	
Female	1,452 (94.8)
Male	80 (5.2)
**Age (years)**	
Mean (Standard Deviation)	46 (23)
<30	1,036 (67.6)
≥30	496 (32.4)
**Educational status**	
None	351 (22.9)
Primary	753 (49.2)
Secondary	314 (20.5)
Tertiary	114 (7.4)
**Employment status**	
Employed	466 (30.4)
Unemployed	1066 (69.6)
Payment for card	
No	961 (62.7)
Yes	571 (37.3)

### Vaccination card retention

**Fig 1:**
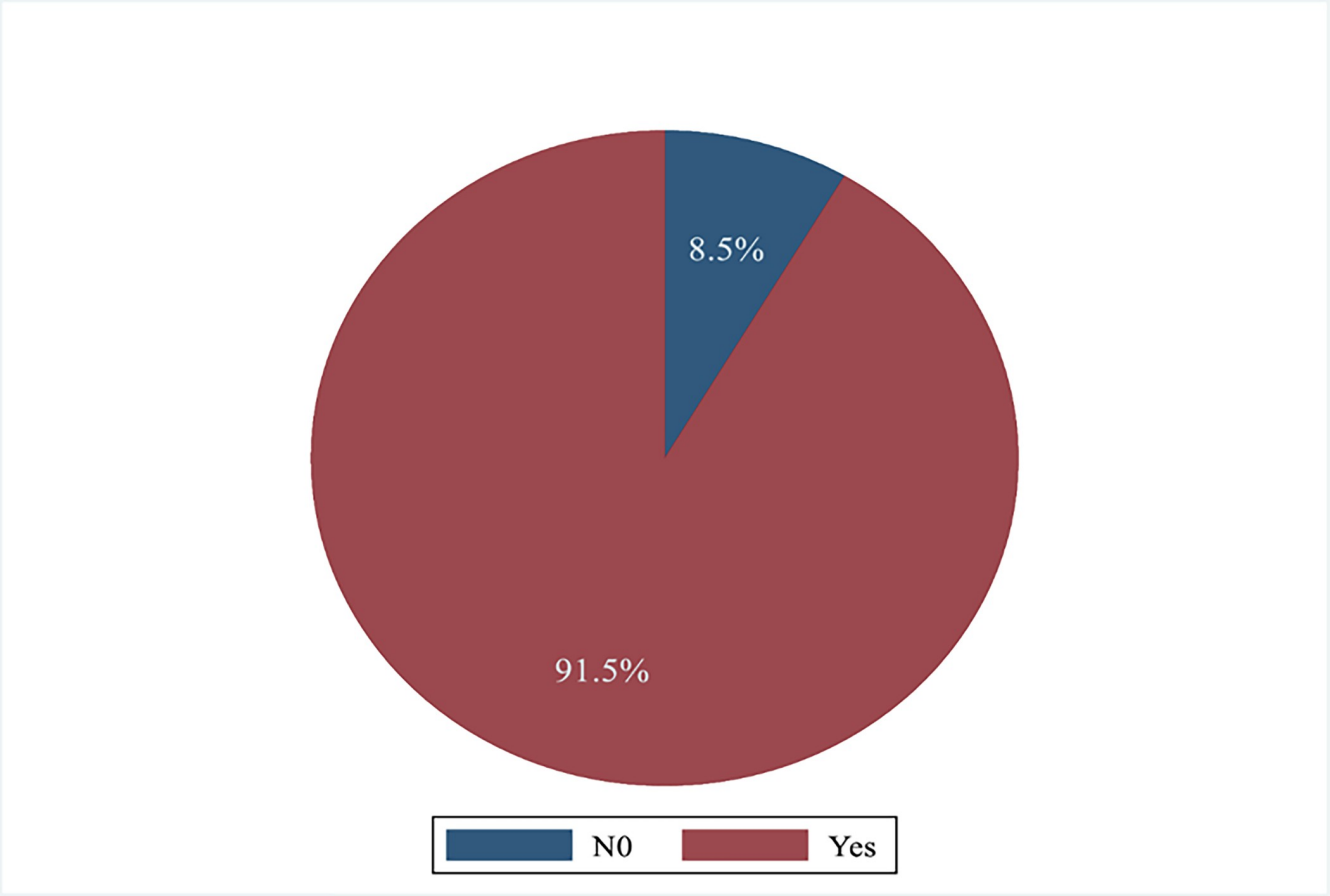
Percentage of caregivers with retained vaccination card in Tamale Metro.

**Fig 2:**
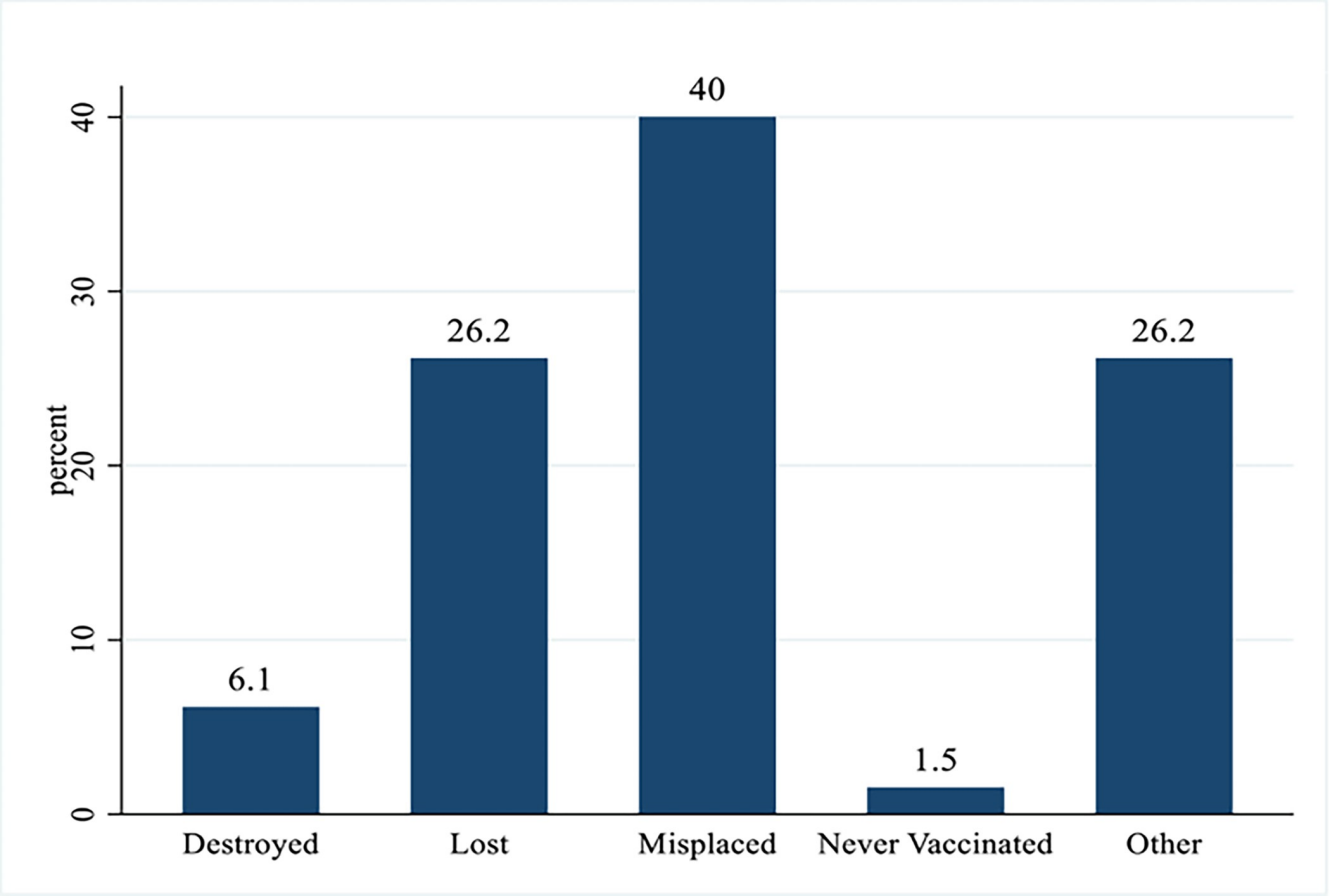
Reasons for not retaining vaccination cards in Tamale Metro.

Of the 1,532 sample, 1,402 (91.5%) presented vaccination cards as proof of vaccination status (Fig 1). Of the remaining 130, misplaced (40%) was the predominantly reportedly reason for non-retention, followed by other reasons such as being locked during the study or feeling lazy to retrieve card (26.2%), and lost (26.2%) (Fig 2).

In a multivariate regression analysis, geographic location, age of child and whether card was paid for or not were significant predictors of vaccination card retention. Living in Tamale Central (AOR = 0.51; 95% CI = 0.29–0.90) or Nyohini (AOR = 0.28; 95% CI = 0.15–0.50) sub-Metros was negatively associated with card retention. Similarly, compared to children aged 0–9 months, those aged 24–59 months had lower odds (AOR = 0.39; 95% CI = 0.22–0.68) of having their vaccination cards retained. However, caregivers who paid for the card had higher odds (AOR = 5.14; 95% CI = 2.95–8.95) of retention (see Table 2).

**Table 2:** Factors associated with vaccination card retention in Tamale Metropolis, Ghana.

	Retention (n = 1,532)		Univariate analysis		Multivariate analysis	
Variable	Yes	No	COR (95% CI)	P value	AOR (95% CI)	P value
**Sub-Metro**						
Bilpela	385	22	Reference		Reference	
Nyohini	311	40	0.44(0.26–0.76)	0.003*	0.28(0.15–0.50)	<0.0001*
Tamale Central	370	49	0.43(0.26–0.73)	0.002*	0.51(0.29–0.90)	0.019*
Vittin	336	19	1.01(0.54–1.90)	0.974	0.91(0.47–1.78)	0.788
**Sex of child**						
Female	662	61	Reference			
Male	740	69	0.99(0.69–1.42)	0.949		
**Age of child (months)**						
0–9	322	19	Reference		Reference	
10–18	297	16	1.09(0.55–2.17)	0.794	0.87(0.42–1.78)	0.702
19–23	137	15	0.54(0.26–1.09)	0.086	0.52(0.24–1.10)	0.086
24–59	646	80	0.48(0.28–0.80)	0.005	0.39(0.22–0.68)	0.001*
**Place of delivery**						
Home/TBA	95	5	Reference		Reference	
Privately owned facility	45	4	1.19(0.22–6.34)	0.843	1.45(0.25–8.22)	0.676
Publicly owned facility	1260	123	0.51(0.22–1.18)	0.187	0.82(0.31–2.13)	0.679
**Age of respondent (years)**						
<30	952	84	Reference			
≥30	450	46	0.86(0.59–1.26)	0.444		
**Sex of respondent**						
Female	1328	124	Reference			
Male	74	6	1.15(0.49–2.70)	0.745		
**Educational status of respondent**						
None	316	35	Reference		Reference	
Primary	702	51	1.52(0.97–239)	0.066	1.27(0.78–2.10)	0.335
Secondary	281	33	0.94(0.57–1.55)	0.819	0.83(0.49–1.42)	0.501
Tertiary	103	11	1.03(0.51–2.11)	0.920	0.96(0.43–2.11)	0.910
**Employment status of respondent**						
Employed	420	46	Reference		Reference	
Unemployed	982	84	1.28(0.88–1.87)	0.199	1.05(0.65–1.68)	0.843
**Relationship of child to respondent**						
Mother	1336	121	Reference		Reference	
Father	54	6	0.82(0.34–1.39)	0.643	1.23(0.45–3.33)	0.684
Other^b^	12	3	0.36(0.10–1.30)	0.120	0.24(0.05–1.25)	0.090
**Pay for card**						
No	848	113	Reference		Reference	
Yes	554	17	4.28(2.54–7.2)	<0.0001*	5.14(2.95–8.95)	<0.0001*

^b^ = auntie/sibling

*p value <0.05; COR = Crude Odds Ratio; AOR = Adjusted Odds Ratio; CI = Confidence Interval; TBA = Traditional Birth Attendant.

## Discussion

Our study shows a high (91.5%) vaccination card retention rate. A study conducted in the Kwahu Afram Plains in the Eastern region of Ghana among 12–23 months old children reported a similar card retention rate of 91.6% [22]. The similarity in findings of the two studies may be explained by the high access to immunization services [22],which provides avenue for reinforcing educational messages about the importance of card retention. Additionally, in most schools in Ghana, presentation of the vaccination card is a requirement for enrolment in school, which also tend to encourage retention. High availability of vaccination cards allows tracking of the individual vaccination history of children and for providing reliable data for epidemiologic decisions. Additionally, the high retention rate has implications on individual children, the health system and general society, especially during vaccination campaigns for children in the immunization program. It helps vaccination service providers to know which vaccines are received and those that are missed. Consequently, interventions aimed at improving vaccination card retention in the study setting may improve timely receipt of vaccine doses [23].Though slightly lower than the findings of our study, two nationally representative surveys in Ghana among children aged 12–23 months reported vaccination card retention rates of 80% in 2014 [16] and 78.5% in 2022 [15]. This slight differences in vaccination card retention rate in the present study and the national surveys may be explained by the fact that our study was carried out in Tamale Metropolis, an urban setting, while the other studies were nation-wide. In urban settings, individuals may have better access to healthcare facilities, making it easier for them to receive vaccinations and retain their vaccination cards [24]. In contrast, However, lower rates of retention have been reported by other studies in Senegal (73.7%) [25], Uganda (66.0%) [26], Cameroon (29.9%) [27], Zimbabwe (68%) [28], and Nigeria (63.3%, 32.6%) [29,30]. The observed discrepancies in retention rates in this study and other studies may be explained by the difference in study settings. While our study was conducted in Tamale Metropolis, an urban setting, the other studies were conducted in rural settings or during national surveys. In remote or rural areas, access to healthcare services may be limited, leading to lower card retention rates. This suggests that vaccination card retention may differ vastly across contexts. Retention rates among urban settlers may be higher than their rural counterparts. However, this requires further investigation to establish the relationship.

Residing in Nyohini or Tamale Central sub-Metros was negatively associated with vaccination card retention. This may be partly explained by the fact that caregivers in these sub-Metros may have low awareness level on the importance of keeping the vaccination card, as this may have not been emphasized by healthcare providers who issued cards. Additionally, the Nyohini and Tamale Central sub-Metros are predominantly slum settlements inhabited by traders. Traders often have competing schedules and as such, have little time to follow their children’s immunization schedule and may occasionally delegate the responsibility of taking children to vaccination centers to others. When a vaccination card is handled by more than one person, it has greater chances of getting lost, which may explain the reason why caregivers resident in Nyohini and Tamale Central had limited odds of retaining vaccination cards. Also, slum populations are often characterized by high levels of transience and informal settlements. When people move frequently, it makes it challenging to maintain consistent healthcare records, including vaccination cards.

Similarly, children aged 24–59 months had reduced odds of having their cards retained. Studies in Senegal [25], Uganda [26] and Pakistan [31] have reported similar findings that caregivers of older children were less likely to retain vaccination cards. The possible reason contributing to this phenomenon is that caregivers may not understand the importance of keeping the card after completing the vaccination schedule. Lack of awareness about the potential need for vaccination records in the future can contribute to neglecting the preservation of these cards. Additionally, if parents perceive the risk of vaccine-preventable diseases to be low among older children, they may not prioritize retaining vaccination cards for these children. However, this creates challenges, particularly for children who have not completed their vaccination schedule and the health system. Without a vaccination card vaccination service providers may not know which vaccines have been given, often leading to repeated doses and consequent vaccine wastage.

Caregivers who paid for the vaccination card had increased odds of retaining it. The possible reason is that caregivers who pay for the vaccination cards may recognize its value and are more likely to take good care of them. On the other hand, they may want to avoid paying for replacement in the event that it is lost. Payment for vaccination cards may introduce an element of self-accountability in vaccine service use to enhance card retention and follow-up vaccinations. However, sale of vaccination cards is against the vaccination policy in Ghana, even in times of shortages, when health facilities print copies to support service delivery. It is expected that the cost of making copies of the vaccination card be borne by the health facilities without transfer to service users.

### Strengths and limitations of the study

This study used a relatively large sample; therefore, the findings can be interpreted in wider context. Additionally, the study used an objective measure through physical inspection to assess card retention. However, findings of this study should be interpreted in the light of the fact that as cross-sectional study, it difficult to establish causality between exposures and vaccination card retention. Additionally, the failure of some of the caregivers to present cards for inspection does not mean that the children do not have them.

## Conclusions and recommendations

Our study found high vaccination card retention rate. Health sub-Metro type of child-caregiver pairs, age of child and payment for card were significantly associated with vaccination card retention.

Health care providers in the Nyohini and Tamale Central sub-Metros should endeavor to intensify education of caregivers on the importance of vaccination cards and the need for safe keeping. Additionally, caregivers of older children should be educated about the importance of keeping cards after the child has completed vaccination schedule. Given that payment for card improves retention, a policy review by the Ghana Health Service is recommended to permit cost sharing to make room for a little charge, not to cover the cost of the card but to enhance retention, particularly in urban settings.

Improving the retention of vaccination cards generally improves the quality of data during surveys and enhances follow up strategies for children in the immunization program.

## Supporting information

S1 Data Database of manuscript: Predictors of vaccination card retention. (DTA)

## Abbreviations

AOR: adjusted odds ratio
BCG: Baccille Calmette Guérin
CHPS: community-based health planning and services
CI: confidence interval
COR: crude odds ratio
DEFF: design effect
DPT: diphtheria, pertussis and tetanus
EPI: expanded program of immunization
HBR: home-based vaccination cards
HepB: hepatitis B
Hib: haemophilus influenzae type b
ICC: intra-cluster coefficient
IPV: inactivated polio vaccine
LMICs: low- and middle-income countries
Men A: Meningococcal A conjugate vaccine
OPV: oral polio vaccine
PCV: Pneumococcal conjugate vaccine 13-valent
TBA: traditional birth attendant
